# Antibacterial and antiviral properties of punicalagin (Review)

**DOI:** 10.3892/mi.2025.264

**Published:** 2025-08-26

**Authors:** Zhuoning Song, Yadong Wang, Peihua Zhang, Ying Wang, Yuan Li, Fang Liu, Jinzhao Long, Haiyan Yang

**Affiliations:** 1 Department of Epidemiology, School of Public Health, Zhengzhou University, Zhengzhou, Henan 450001, P.R. China; 2 Department of Toxicology, Henan Center for Disease Control and Prevention, Zhengzhou, Henan 450016, P.R. China

**Keywords:** punicalagin, antibacterial, antivirus, biofilm, drug resistance, mechanism

## Abstract

Punicalagin, a polyphenolic compound extracted from pomegranate peel, has received increasing attention in recent years due to its antibacterial and antiviral properties. Punicalagin is capable of inhibiting bacterial growth at sub-inhibitory concentrations by affecting cell membrane formation, disrupting membrane integrity, altering cell permeability, affecting efflux pumps, interfering with quorum sensing and influencing virulence factors. Additionally, punicalagin inhibits viruses by modulating enzyme activity, interacting with viral surface proteins, affecting gene expression, blocking viral attachment, disrupting virus receptor interaction and inhibiting viral replication. Notably, it exerts synergistic effects on bacterial infections in combination with certain antibiotics, such as oxacillin, particularly against methicillin-resistant *Staphylococcus aureus*. However, the wide variation in the minimum inhibitory concentrations (MICs) of punicalagin against various bacterial strains, in addition to the lack of safety, toxicity, or side-effect profile data, underscores the need for additional studies. The present review discusses the advances in the antibacterial and antiviral activities of punicalagin, with in-depth analyses of its mechanisms of action and potential applications in combating microbial infections. Future research is required to focus on addressing the variability in reported MICs, optimizing their bioavailability and conducting preclinical studies to provide a new paradigm for the antimicrobial applications of natural polyphenols.

## 1. Introduction

Recently, the increasing prevalence of drug-resistant pathogens has created a strong demand for new antibiotics, further spurring the active exploration of natural plant compounds as alternatives to synthetic antimicrobial agents (1-3). Plants provide an abundance of antimicrobial materials, including tannins (4), flavonoids (5), phenolic acids (6), essential oils (7), saponins (8), alkaloids, terpenoids (9), coumarins (10) and lignans (11). Among these, polyphenols including punicalagin have garnered significant attention due to their diverse chemical structures and multiple mechanisms of action.

Punicalagin [2,3-(S)-hexahydroxydiphenoyl-4,6-(S,S)-gallagyl-D-glucose] is one of the principal active tannins isolated from pomegranate peel. Its molecular formula is C_48_H_28_O_30_, with a molecular weight of 1084.72(12). It is soluble in polar solvents, such as water and ethanol. In addition, the compound contains multiple phenolic hydroxyl groups, the presence of which renders punicalagin susceptible to degradation in alkaline environments. This results in the generation of compounds, such as andrographolide and ellagic acid, which possess the physicochemical properties of hydrolysable tannins. Additionally, the variety of ring structures (e.g., benzene rings) and chemical bonds in its structure imparts flexibility and conformational adaptability to the molecule, which may influence its biological activity.

Punicalagin exhibits a wide range of biological and pharmacological activities (13-20). Compared to other polyphenols, punicalagin exhibits unique advantages, such as its diverse mechanisms of action against both bacteria and viruses, and it has the potential to disrupt biofilm formation and quorum sensing, rendering it a promising candidate for developing new antimicrobial agents (21-23). Previous studies have mainly focused on its antioxidant (24,25), anticancer, anti-inflammatory and chronic-disease-relieving properties (26-28). In addition, punicalagin has been shown to have the ability to combat both bacterial and viral pathogens (29-32). While recent *in vivo* research confirms its therapeutic potential against bacterial infections (33), clinical evidence remains limited. Thus far, to the best of our knowledge, no comprehensive review has been published on this topic. Accordingly, the present review discusses the antibacterial and antiviral efficacy of punicalagin. The present review aimed to provide a comprehensive summary of the potential opportunities and challenges encountered in developing punicalagin as an effective therapeutic agent.

## 2. Antibacterial effects of punicalagin

**Figure 1:**
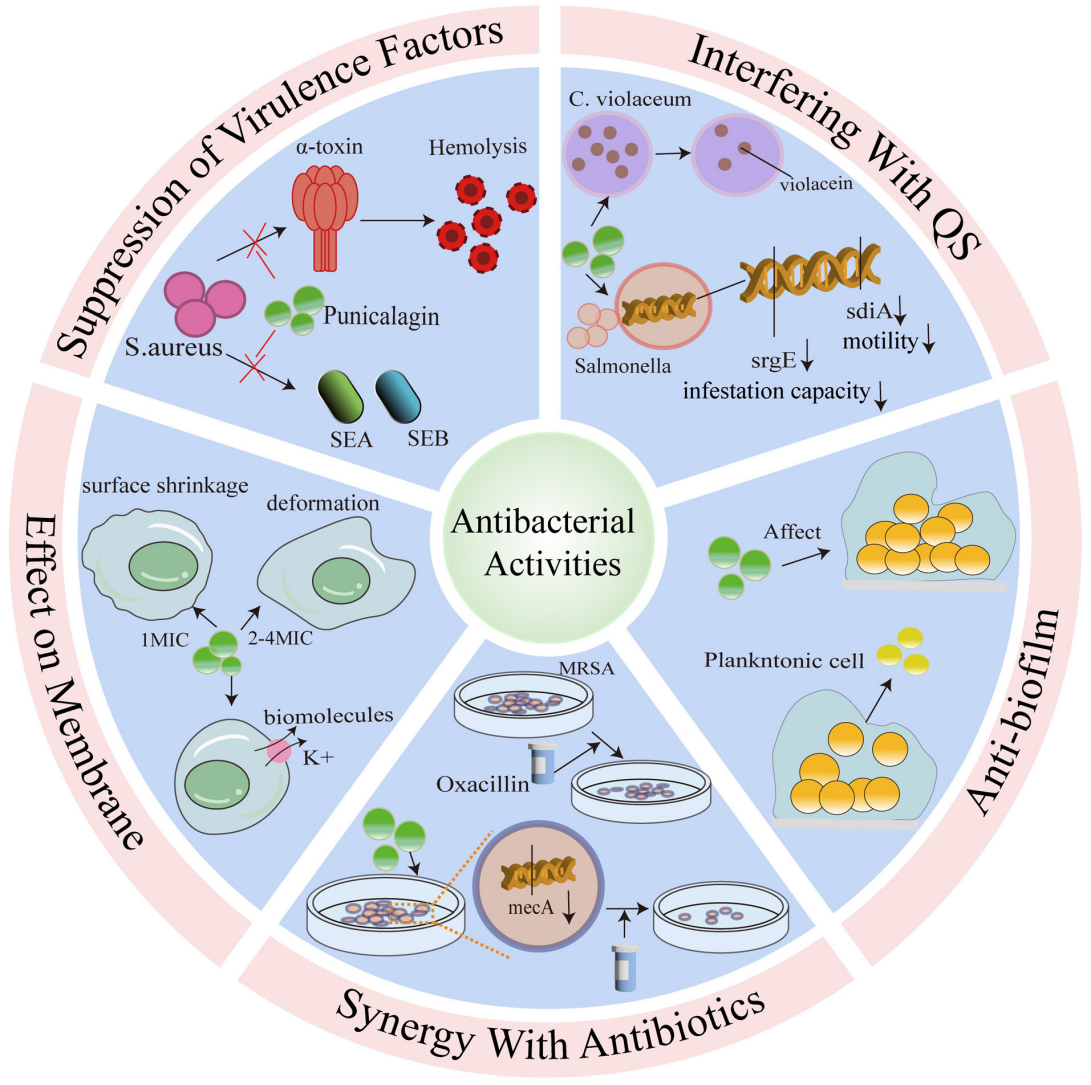
Antibacterial activities of punicalagin. MIC, minimum inhibitory concentration; MRSA, methicillin-resistant *Staphylococcus aureus*; SEA, staphylococcal enterotoxin A; SEB, staphylococcal enterotoxin B.

As illustrated in Fig. 1, the mechanisms of action of punicalagin against bacteria include targeting bacterial cell membranes and biofilms, quorum sensing, suppressing virulence factors and exerting synergistic effects with antibiotics.

### Antibacterial activity

The inhibitory effects of punicalagin on bacterial growth have been extensively investigated, and its antimicrobial activity is usually measured using the minimum inhibitory concentration (MIC). A summary of the studies on the antibacterial effects of punicalagin against different bacteria is provided in Table I (34-38). Data from the literature reveal that MIC values for different bacterial strains vary significantly depending on the bacterial strains (34-38).

However, for the same strains, there were significant discrepancies in the reported MIC values. These variances are likely due to the following factors: i) Inherent differences between strains; ii) differences in culture conditions (e.g., Mueller-Hinton agar vs. tryptic soy broth); and/or iii) a discrepancy in the assay method (e.g., two-fold dilution method vs. agar dilution method). For instance, the MIC value of punicalagin against *Escherichia coli* ATCC 25922 was reported to be 1.2 µg/ml (35), while another study indicated an MIC value of 160,000 µg/ml for the same strain (36). Similarly, notable discrepancies were found in the MIC values for *Staphylococcus aureus* (*S. aureus*) ATCC 29213, with one study reporting an MIC value of 250 µg/ml (38) and another study reporting 20,000 µg/ml (36).

In addition, the antibacterial and bactericidal effects of punicalagin can be observed through bacterial growth curves. For instance, at MIC levels, punicalagin was previously shown to exert no significant effect on the growth of *Salmonella typhimurium* (*S. typhimurium*) within the first 2 h following exposure. However, by 24 h, viable cell counts were reduced by ~2 log units (39). For treatments at 2x and 4x MIC levels, the growth of *S. typhimurium* was completely inhibited within 24 h, indicating a bactericidal effect. Similarly, Xu *et al* (38) reported that at certain MIC levels, punicalagin decreased ~0.5 log of viable *S. aureus* cells following 2 h of exposure, and ~1 log of these cells was reduced following 24 h of incubation, indicating a bacteriostatic effect. When *S. aureus* was treated with 2x and 4x the MIC of punicalagin, the loss of viable cells was significantly more pronounced immediately after initial exposure (38).

Overall, punicalagin has demonstrated antibacterial effects against multiple bacterial strains *in vitro*; however, limited *in vivo* studies are available. Further studies are thus required to elucidate the differential susceptibility of various bacterial strains to punicalagin and to perform *in vivo* animal testing, which is crucial for the development of antibacterial drugs.

### Inhibition of bacterial virulence factors of punicalagin

A key aspect of microbial pathogenesis is virulence, which is the ability of a pathogen to produce disease in a host, and virulence factors are the mechanisms through which pathogens cause damage to the host. These mechanisms include the secretion of toxins, adhesion to host surfaces, invasion of host tissues and the formation of biofilms (40). Bacterial pathogens can secrete diverse toxins with different structures and functions, which are crucial for the development of infectious diseases (41,42). Punicalagin is notable for its ability to directly target N-acyl-homoserine lactone-dependent quorum sensing (QS) systems, which are associated with virulence, invasiveness and pathogenicity. Staphylococcal protein A (SpA) is a key virulence factor of *S. aureus*, which binds to immunoglobulins through its Fcγ domain and the Fab heavy chain of V_H_3 family antibodies, thereby interfering with bacterial opsonophagocytosis and the development of adaptive B cell responses (43,44). Punicalagin reduces the fixation of SpA to the bacterial cell wall by inhibiting SrtA (33). Furthermore, punicalagin inhibits the expression of major virulence factors, such as hemolysin and enterotoxin in methicillin-resistant *S. aureus* (MRSA) (45). The main virulence factors of *S. aureus* cover a wide range of antigens, enzymes, cytotoxins and exotoxins. Among the virulence factors secreted by *S. aureus*, α-toxin is the main component of its hemolytic activity, while enterotoxin is one of the most prominent exotoxins of the bacterium. Enterotoxin is able to function as a superantigen to activate T-cells and induce them to release large amounts of pro-inflammatory cytokines (46). Therefore, targeting these virulence factors may provide an ideal approach for developing antibacterial therapeutics against *S. aureus* infections. Sub-inhibitory concentrations of punicalagin have been shown to reduce the production of α-toxin, staphylococcal enterotoxin A and staphylococcal enterotoxin B in an MRSA in a concentration-dependent manner and decrease the activity of the culture supernatant of *S. aureus* induced by tumor necrosis factor (45). Additionally, Li *et al* (37) found that punicalagin downregulated the expression of the majority of selected virulence genes in *Salmonella* and significantly reduced the invasion of *Salmonella* into colon cells, although it had no effect on adhesion. Moreover, sub-inhibitory concentrations of punicalagin have been shown to reduce the colony-forming ability and virulence factor expression of *S. typhimurium* (30). These findings strongly indicated that punicalagin might be used as a novel therapeutic agent in the treatment of bacterial infections.

### Anti-biofilm properties of punicalagin

Bacteria can exist as planktonic cells, or they can attach to surfaces to form aggregates, which are termed biofilms. In biofilms, bacteria produce extracellular material in which cells are embedded (47). The formation of microbial biofilms enables individual planktonic cells to adopt a multicellular growth pattern (48). The benefit of bacterial biofilm formation is the creation of a more stable environment that allows bacteria to resist environmental threats, such as phagocytosis and antimicrobial agents (49). Thus, the inhibition of biofilm formation is viewed as a promising method for controlling bacterial infections (50). Punicalagin has been investigated for its ability to reduce biofilm formation and to affect the normal function of biofilms in various bacterial pathogens, and it exerts a significant inhibitory effect on the biofilm formation of *S. aureus*. In a previous study, the biomass of biofilm was reduced to 42.0% when the concentration of punicalagin was 1/64 MIC compared to the control, and it was significantly reduced to 8.1% when the concentration was further increased to 1/32 MIC (51). Similarly, the study by Xu *et al* (38) demonstrated that the biofilm formation of *S. aureus* was inhibited by 47% when it was treated with punicalagin at 1/64 MIC, while biofilm formation was inhibited by >90% when the concentration exceeded 1/32 MIC. Additionally, the study by Song *et al* (33) demonstrated that punicalagin effectively reduced biofilm formation by inhibiting sortase A activity on the cell surface of *S. aureus*.

### Effects of punicalagin on the cytoplasmic membrane

The cytoplasmic membrane is a common target of action for a number of antimicrobial agents, and cell viability is usually closely linked to the integrity of the cytoplasmic membrane. It is generally recognized that the antimicrobial activity of numerous compounds is partly due to their ability to selectively disrupt the membrane structure and function of a variety of microorganisms. The significant loss of cytoplasmic components implies irreversible damage to the cytoplasmic membrane.

Potassium ions (K^+^) are the major cytoplasmic cations essential for bacterial growth, and they are involved in several key biological functions. Excessive K^+^ efflux can affect bacterial growth and can even result in mortality (52). The study by Liu *et al* (34) demonstrated that treatment with punicalagin induced significant potassium efflux from *Vibrio parahaemolyticus* (*V. parahaemolyticus*) bacterial cells in a concentration-dependent manner. In addition to ions, punicalagin increased the permeability to biomolecules such as nucleic acid. As the concentration of punicalagin treatment increased, the integrity of the *V. parahaemolyticus* cell membrane decreased and the severity of cellular damage increased in a concentration-dependent manner (34). This was mainly characterized by cell surface shrinkage under a 1x MIC treatment and extensive cellular deformation; the disruption of the cell membrane; and cytoplasmic leakage under 2x and 4x MIC treatments (44). Similarly, another study (38) demonstrated that punicalagin induced an immediate and massive efflux of K^+^ from *S. aureus*, suggesting that punicalagin affects the cytoplasmic membrane by increasing its permeability or interfering with the transmembrane proton gradient. Following treatment with punicalagin, *S. aureus* cells exhibited significant enlargement and a roughened surface, with a higher degree of deformation observed following treatment with high concentrations of punicalagin (38).

Moreover, it has been reported that punicalagin can exert an inhibitory effect on *Salmonella* by disrupting the cell membrane, as evidenced by potassium leakage, membrane depolarization, an increased pH gradient and microstructural alterations (39).

### Punicalagin interference with QS

QS systems enable bacteria to respond to cell density and regulate gene expression through cell-to-cell communication (53). Bacteria with QS produce and release signaling molecules, termed autoinducers, that increase cell density (54). When the concentration of autoinducers reaches a certain threshold, the bacteria detect this signal and trigger changes in gene expression. In bacterial pathogens, QS systems play a crucial role in regulating virulence gene expression, allowing bacteria to launch coordinated attacks to overwhelm host defenses (55,56). QS systems have recently gained increasing attention as attractive targets for antimicrobial therapy (57). It has been suggested that the inactivation of the QS systems of a pathogen can result in a significant reduction in its virulence (58). In the study by Li *et al* (37), the findings from quantitative QS inhibition assays revealed that punicalagin inhibited the expression of *Salmonella* motility-related genes (e.g., sdiA and srgE), and punicalagin exhibited concentration-dependent anti-QS activity against *Chromobacterium violaceum*, suppressing violacein production to ~94.56% of the control at 1/64 MIC and markedly reducing it to 64.66% of the control at 1/32 MIC. However, research on the effects of punicalagin on bacterial QS systems remains limited, and further studies are warranted to fully elucidate its potential as an antimicrobial agent.

### Synergistic effects of punicalagin with antibiotics against bacteria

Antibiotic adjuvants are used to achieve two primary objectives: i) Expand the antimicrobial spectrum of antibiotics; and ii) combat multidrug-resistant (MDR) bacterial infections through synergy with antibiotics. Therefore, the rational use of combination therapy with two or more drugs, such as antibiotics and natural compounds, can enhance antimicrobial efficacy by curbing the development of resistance in MDR bacteria (59-61). Punicalagin, in addition to its standalone antimicrobial activity, also exerts synergistic antimicrobial effects when combined with several common antibiotics. It is an effective enhancer, increasing the potency of cefotaxime and oxacillin against Gram-positive bacteria by interfering with bacterial transcription mechanisms and acting as a virulence inhibitor (33,62). In addition, the potential mechanism by which punicalagin enhances the potency of antibiotics against Gram-negative bacteria may involve membrane damage, allowing greater antibiotic absorption and toxicity. Additionally, impaired efflux pumps can lead to fatal interactions, rendering bacterial cells more sensitive to accelerated drug-induced cell death (39,63). The study by Song *et al* (33) demonstrated the synergistic effect of punicalagin with cefotaxime and ceftriaxone sodium, providing improved protection against MRSA-induced fatal pneumonia in mice, with inhibitory concentration fractional index values of 0.28125 and 0.3125, respectively. In another study, punicalagin enhanced the efficacy of oxacillin against MRSA by downregulating the transcription of *mecA* (a gene marker of methicillin resistance), resulting in a decrease in penicillin-binding protein 2a levels (62). In the presence of punicalagin, the MIC of oxacillin was reduced by 4- to 64-fold, clearly indicating that punicalagin enhanced the susceptibility of MRSA to oxacillin. This suggested that combination therapy with these two drugs could reverse β-lactam resistance in MRSA (62). Furthermore, it has also been demosntrated that punicalagin can enhance the sensitivity of MRSA to oxacillin (30).

In summary, punicalagin enhances the susceptibility of bacteria to some conventional antibiotics. However, whether punicalagin in combination with antibiotics enhances the antimicrobial effect on a broader range of bacteria remains to be studied.

## 3. Antiviral effects of punicalagin

The continued rise in the number of global viral infections has become a pressing issue for public health, and the development of new drugs is urgently required. In particular, coronavirus disease 2019 (COVID-19), caused by severe acute respiratory syndrome coronavirus 2 (SARS-CoV-2), has imposed an immense burden on global health, economies and societies (64-66). In this context, punicalagin has emerged as a promising antiviral agent against SARS-CoV-2. It has also been shown to inhibit a range of viruses, including human cytomegalovirus (HCMV), herpes simplex virus (HSV-1), hepatitis C virus (HCV), respiratory syncytial virus (RSV), measles virus (MV) and dengue virus (DENV), without exhibiting significant cytotoxicity at various concentrations (67).

### Antiviral activity

The antiviral ability of punicalagin has been extensively studied by measuring the 50% inhibitory concentration (IC_50_), 50% effective concentration (EC_50_), 50% cytotoxic concentration (CC_50_), and selectivity index (SI). A summary of the data on the activity of punicalagin against various viral species is presented in Table II (68-77). The data obtained from the literature demonstrated that there are significant differences in IC_50_, EC_50_ and CC_50_ values, depending on the strain of viruses and substances (68-77).

### Respiratory viruses

In recent years, respiratory infectious diseases have occurred more frequently worldwide, posing a serious threat to human health. Among these diseases, those caused by viral infections account for a large proportion. There are eight main viruses that cause acute respiratory infections in humans: RSV, influenza virus, coronavirus, rhinovirus, parainfluenza virus, adenovirus, boca virus and metapneumovirus (78). Research indicates that punicalagin can effectively eliminate infections caused by HCMV, HCV, DENV, MV and RSV at micromolar concentrations in a dose-dependent manner without significant cytotoxicity (67).

**Figure 2:**
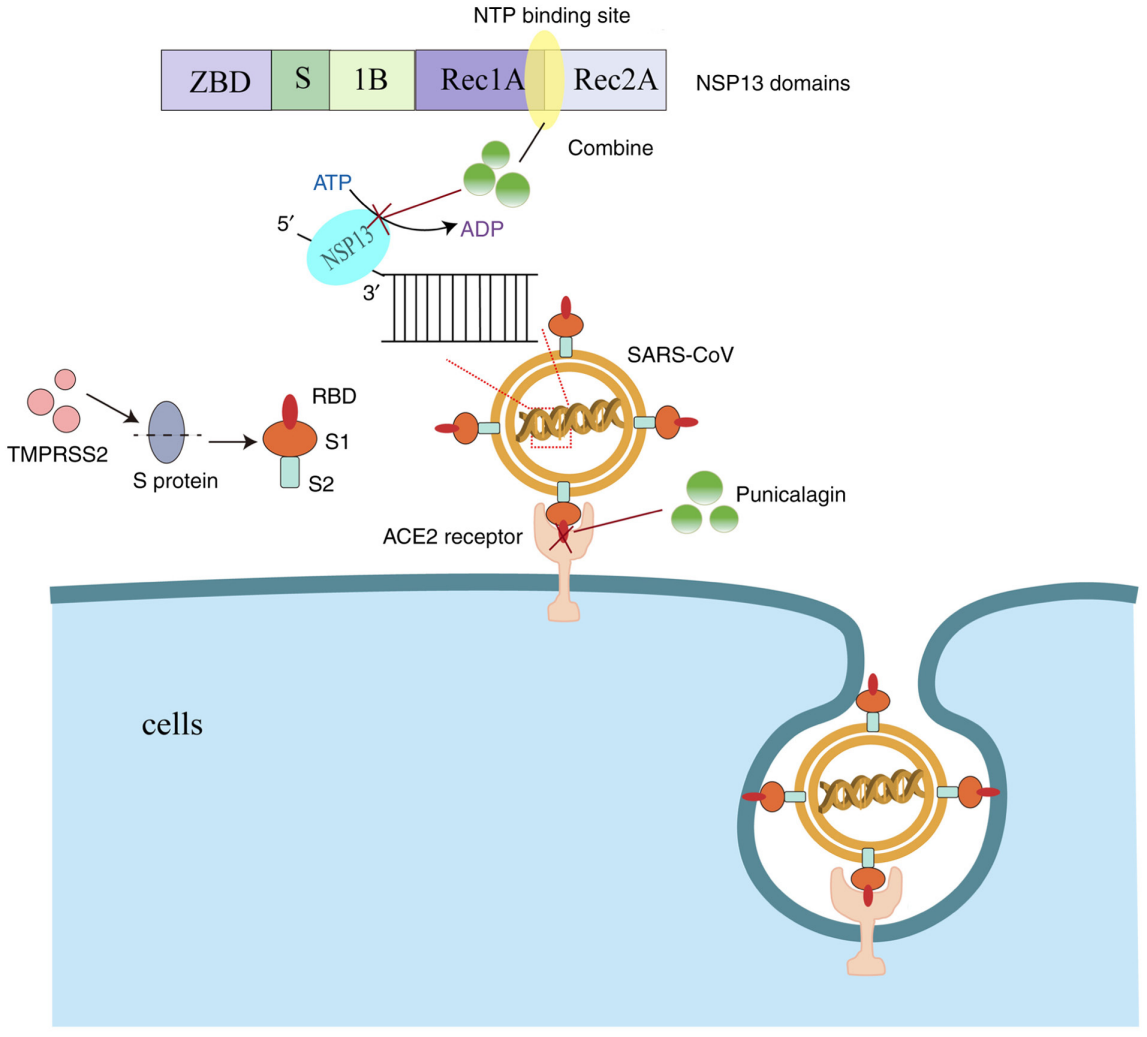
Punicalagin disrupts the interaction between the spike glycoprotein RBD and the ACE2 receptor and affects NSP13 helicase. RBD, receptor-binding domain; ACE2, angiotensin-converting enzyme 2; TMPRSS2, transmembrane protease serine 2.

Infections with SARS-CoV-2 begin when the virus enters the cell through interactions between the viral spike (S) protein and host cell surface receptor angiotensin-converting enzyme 2 (ACE2) and transmembrane protease serine 2 (TMPRSS2), initiating the S protein (79). TMPRSS2 proteolytically processes the S protein into S1 and S2 fragments. The S1 fragment interacts with the ACE2 receptor via its receptor-binding domain (RBD), while the S2 fragment promotes the fusion of the virus with the host cell membrane (80). Punicalagin has been shown to disrupt the interaction between the spike glycoprotein RBD and the ACE2 receptor, with an IC_50_ value of 29 µM (68,81). SARS-CoV-2 NSP13 helicase is an essential enzyme for viral replication, and it has been identified as an ideal target for the development of antiviral drugs (82). Punicalagin binds NSP13 directly on the interface between the 1A and 2A domains and overlaps with the triphosphate binding site of the NTP, suggesting a direct competition mechanism. Punicalagin produces an antiviral effect by inhibiting the hydrolysis of ATP by NSP13 and the binding of NSP13 to DNA substrates, affecting viral replication (69). The aforementioned two mechanisms are illustrated in Fig. 2. Moreover, the main protease of SARS-CoV-2, also known as 3-chymotrypsin-like protease (3CLpro) or main protease (Mpro), is the only cysteine protease found in coronaviruses, and it is essential for viral replication (83,84). Published biological studies have demonstrated that small molecules that inhibit the activity of SARS-CoV-2 Mpro can lead to the inhibition of viral RNA replication, thereby exerting an antiviral effect (85-87). The IC_50_ value of punicalagin against 3CL protease has been found to be 6.192 µg/ml (70). In another similar experimental study, the results revealed that punicalagin reduced the activity of Mpro in a dose-dependent manner, with an IC_50_ value of 4.62±0.27 µM (71). Taken together, these studies suggest that punicalagin can exert anti-SARS-CoV-2 effects by the following mechanisms: i) Disrupting the interaction of spike glycoprotein RBD with ACE2 receptors; ii) inhibiting NSP13 helicase; and iii) Mpro activity.

Seasonal influenza is characterized by high morbidity and mortality rates, and it is mainly caused by the influenza A virus (IAV) or influenza B virus (IBV) (88). Due to antigenic mutations, adaptations, rearrangements and highly virulent strains, IAV can cause a devastating pandemic (89). In the search for natural products, punicalagin was previously shown to inhibit the replication of recombinant IAV PR8-PB2-Gluc (90,91). As previously demonstrated, punicalagin inhibited PR8-PB2-Gluc replication in a dose-responsive manner, with an IC_50_ value of 1.25±0.06 µM (75), and it induced a significant dose-dependent decrease in viral titers (76). In addition, the results from the virus release inhibition test revealed that punicalagin could block IAV release (75). Punicalagin also exhibited virucidal activity and inhibited the agglutination of the influenza virus to red blood cells (76). Punicalagin has been shown to inhibit the proliferation of IAV under both multi-cycle and single-cycle growth conditions (92).

In summary, some studies (as aforementioned) have shown that punicalagin inhibits a variety of respiratory viruses *in vitro*. These findings highlight the need for further clinical studies to explore the therapeutic and preventive potential of punicalagin in treating diseases caused by respiratory viruses (75).

### Enterovirus

Globally, hand, foot and mouth disease (HFMD) is a common illness among young children and is a highly prevalent infectious disease in children <5 years of age (93). Coxsackievirus A16 (CVA16) and enterovirus 71 (EV71) are the two main pathogens of HFMD (94). Punicalagin has been shown to reduce the cytopathic effects caused by CAV16 infections in a dose-dependent manner. It inhibits infections by targeting the early entry phase of CVA16 via the direct inactivation of cell-free CVA16 viral particles and blocking viral attachment to the host cell surface (95). Previous studies have described punicalagin as a competitive inhibitor of glycosaminoglycans (GAGs), which are involved in viral entry. Punicalagin exerts broad-spectrum antiviral activity by preventing the entry of various enveloped viruses that utilize cell surface GAGs for infection (67), and the necessity of GAG involvement in CVA16 infections has also been demonstrated. The inhibitory effect of punicalagin on CVA16 is attributed to its ability to compete with cell surface GAGs for binding to viral particles, similarly to the action of soluble heparin (96). In addition to its effects on CVA16, punicalagin has also shown promise in combating EV71, another major pathogen associated with HFMD. Researchers have found that punicalagin significantly inhibits EV71 infections in rhabdomyosarcoma cells and reduces mortality in mice following a fatal EV71 challenge by inhibiting viral replication (97).

Therefore, given its demonstrated antiviral activity against both CVA16 and EV71, the two main causative agents of HFMD, punicalagin warrants further exploration and development as a potential therapeutic agent for the treatment of HFMD.

### Hepatitis virus

The most common cause of viral hepatitis is HBV, which is the leading cause of end-stage liver disease worldwide (98). Covalently closed circular DNA (cccDNA) plays a crucial role in the life cycle of HBV. Thus, there is a pressing need to continuously develop new drugs for the treatment of HBV infections, in addition to developing drugs with the ability to eradicate cccDNA from HBV-infected hepatocytes (99,100). In a previous study, punicalagin significantly decreased the production of secreted HBeAg and cccDNA in a dose-dependent manner, without significant alterations in viral DNA replication. However, punicalagin exerted no significant effects on pronuclear/nuclear promoter activity, pgRNA transcription, core protein biosynthesis, or HBsAg secretion (101). Although vaccination is effective in interrupting vertical transmission and provides protection against HBV infections for 90% of the healthy population, there is a lack of 100% effective antiviral treatment options for patients with chronic hepatitis B (102). Thus, the anti-HBV infection effect of punicalagin is worth studying.

### Other viruses

Feline herpesvirus type 1 (FHV-1), a key member of the α-Herpesviridae family, has triggered an extensive epidemiologic burden in feline populations worldwide, and its infection can result in acute upper respiratory syndrome, corneal ulcers, and lethal viral pneumonia; moreover, it now poses a threat to feline health worldwide (103). The complex glycoprotein system on the surface of the viral envelope is a key target for mediating host cell invasion and immune regulation, and 12 functional glycoproteins including glycoprotein B (gB), gC, gD, gE, gG, gH, gI, gJ, gK, gL, gM and gN have been identified (72,104). Among these, gB is an essential glycoprotein for herpesvirus-infected cells, and it plays critical roles in viral adsorption, mediating membrane fusion and in the infection of host cells by the virus (105). As an effective antiviral compound, punicalagin inhibits the early invasion process of the FHV-1 virus through a dual mechanism: i) It may function as a competitive inhibitor, blocking the binding of the viral gB protein to the host cell surface receptor; and ii) it durably inhibits its mediated membrane fusion by irreversibly inducing the closed conformation of the gB protein (72). Similarly, African swine fever (ASF) is an acute febrile infectious disease caused by the African swine fever virus (ASFV), which is characterized by extreme contagiousness and high lethality (106). Punicalagin has been found to act on the early stages of ASFV replication, including attachment and internalization, and it can directly inactivate the virus. In addition, punicalagin can regulate the NF-κB/STAT3/NLRP3 signaling pathway, thereby alleviating ASFV-induced inflammation (107).

Human immunodeficiency virus (HIV) primarily targets CD4^+^ T-lymphocytes and CD8^+^ T-lymphocytes, which are key components of the adaptive immune system; for this reason, HIV infections cause damage to the immune system. Damage to the body occurs at all stages of HIV infection, and patients with acquired immunodeficiency syndrome (AIDS) have a relatively high rate of disability (108,109). While antiretroviral therapy has significantly increased the life expectancy of patients with HIV, the disease remains a major global public health challenge (110). Punicalagin was previously found to inhibit HIV-1 reverse transcriptase-associated RNase H activity and integrase LEDGF-dependent activity, particularly targeting HIV-1 integrase LEDGF-dependent activity (73). This compound effectively suppressed HIV-1 replication in infected H9 lymphocytes while exhibiting low cytotoxicity (73).

Mayaro virus (MAYV) is an emerging arthropod-borne virus (Arbovirus). Mayaro fever (MF), caused by MAYV, typically presents as a non-specific febrile illness that can evolve into an arthritic condition that persists for months after the infection has resolved (111). An anti-MAYV study demonstrated that the ethanolic extract of *Punica granatum* exhibited a high selectivity index value of 49, with significant virucidal activity of about 98%; in contrast, it was observed that a partial selectivity index of 15 with punicalagin as the main component exhibited strong antiviral activity (74).

## 4. Conclusions and future perspectives

With the emergence and evolution of bacterial and viral drug resistance, the potential benefits and therapeutic value of punicalagin are of immense interest to scientists. The present review indicates that punicalagin exerts a broad inhibitory effect on a wide variety of bacteria and viruses. In terms of antibacterial effects, it is able to effectively inhibit the growth of Gram- and Gram-negative bacteria. The inhibitory effects of punicalagin on bacteria are mainly achieved by influencing virulence factors, interfering with the formation and normal activity of biofilms, and disrupting QS. In terms of antiviruses, punicalagin inhibits viral replication and spread by targeting the interaction of viral glycoproteins with host cells and preventing viruses from entering cells. For example, punicalagin exhibits good antiviral effects against both HSV-1 and EV71. In addition, punicalagin also exerts antioxidant and immunomodulatory effects, further enhancing its potential application value in disease prevention and treatment.

Possible interactions with different types of conventional antimicrobial agents also need to be considered in practical applications. Punicalagin, as an effective enhancer, can increase the potency of cefotaxime and oxacillin against Gram-positive bacteria and can function as a virulence inhibitor by interfering with bacterial transcriptional machinery. Therefore, future studies are warranted to explore the potential of using punicalagin as a synergist in combination with other treatments.

It is worth noting that while the present review highlights the multi-targeted antibacterial mechanisms of punicalagin, the risk of bacterial or viral resistance remains unaddressed. Unlike traditional antimicrobial drugs that target a single key pathway, pomegranate peel glycosides exhibit multifaceted pharmacological effects. For example, they inhibit the SARS-CoV-2 NSP13 helicase, 3CLpro protease, and the interactions between the spike protein and ACE2 receptor. These multifaceted effects may create greater evolutionary barriers to microbial escape. However, to draw definitive conclusions, future studies are required to characterize resistance mechanisms through mutation frequency analyses, antimicrobial screening experiments, and comparative risk assessments with traditional drugs. Additionally, the potential risks of punicalagin with respect to bacterial and viral resistance need to be evaluated. Furthermore, future studies are required to further uncover the mechanism of interactions between punicalagin and pathogens, particularly its specific targets at the cellular and molecular levels. On this basis, through chemical modification or nanotechnology and other means, the bioavailability and stability of punicalagin will be improved, its antimicrobial and antiviral efficacy *in vivo* will be enhanced, and new antibacterial and antiviral drug preparations based on punicalagin, such as oral preparations, injections, or topical drugs, will be developed. In addition, no clinical trials or regulatory filings for punicalagin as an antimicrobial have yet been reported. The primary challenges in translating preclinical research findings into clinical applications may include the need for further research to improve their bioavailability and stability, the necessity of conducting comprehensive pharmacokinetic and pharmacodynamic studies, and the requirement of thorough safety assessments to ensure their suitability for human use. It is also critical to conduct clinical trials in order to verify the safety and efficacy of punicalagin in the treatment of bacterial infections and viral infectious diseases and to explore the combined application of punicalagin with other drugs to produce synergistic antibacterial and antiviral effects and reduce the development of drug resistance.

## Figures and Tables

**Table I:** Summary of the antimicrobial properties of punicalagin against various strains of bacteria.

Bacteria	Identifier	MIC (µg/ml)	Cultivation	Test method	(Refs.)
*Vibrio parahaemolyticus*	ATCC17802	200	TSB	Broth microdilution method	(34)
	ATCC 33847	150	TSB	Broth microdilution method	(34)
	RIMD2210633^Sm^	200	TSB	Broth microdilution method	(34)
*Escherichia coli*		>10,000	LB	Broth microdilution method	(30)
	ATCC 25922	1.2	MHA	Agar dilution method	(35)
	ATCC 25922	160,000	NA	Two-fold dilution method	(36)
	IFO 3972	320,000	NA	Two-fold dilution method	(36)
*Salmonella typhi*		>10,000	NA	Two-fold dilution method	(36)
*Salmonella typhimurium*		>10,000	NA	Two-fold dilution method	(36)
*Candida albicans*	ATCC 10231	1.2	MHA	Agar dilution method	(35)
*Pseudomonas aeruginosa*	ATCC 9027	0.60	MHA	Agar dilution method	(35)
*Salmonella*	ATCC 9270	80,000	NA	Two-fold dilution method	(36)
	ATCC 13314	80,000	MHA	Agar dilution method	(36)
	44-1	500	LB	Broth microdilution method	(37)
*Salmonella enterica*	LC 216	>2.4	MHA	Agar dilution method	(35)
*Staphylococcus epidermidis*	ATCC 12228	0.60	MHA	Agar dilution method	(35)
*Staphylococcus xylosus*	LC 57	0.60	MHA	Agar dilution method	(35)
*Staphylococcus aureus*	ATCC 6538	0.60	MHA	Agar dilution method	(35)
	ATCC 29213	250	MHA	Agar dilution method	(38)
	ATCC 29213	20,000	NA	Two-fold dilution method	(36)
	IFO 12732	10,000	NA	Two-fold dilution method	(36)
*Bacillus cereus*	CIP 78.3	>2.4	MHA	Agar dilution method	(35)
*Lactobacillus sakei ssp. Sakei*	ATCC 15521	0.60	MHA	Agar dilution method	(35)
*Lactobacillus plantarum*	CECT 4185	>2.4	MHA	Agar dilution method	(35)
*Pediococcus acidilactici*	LCp1	0.60	MHA	Agar dilution method	(35)
*Enterococcus faecium*	DSMZ10663	>2.4	MHA	Agar dilution method	(35)
*Enterococcus faecalis*	ATCC 19433	0.60	MHA	Agar dilution method	(35)
*Enterococcus mundtii*	LC E23	0.30	MHA	Agar dilution method	(35)
*Enterococcus sulfureus*	LC E28	0.60	MHA	Agar dilution method	(35)
*Enterococcus casseliflavus*	LC E1	0.30	MHA	Agar dilution method	(35)
*Enterococcus columbae*	LC E2	0.60	MHA	Agar dilution method	(35)

MIC, minimum inhibitory concentration; TSB, tryptic soy broth; LB, Lauria-Bertani broth; MHB, Mueller-Hinton broth; MHA, Mueller-Hinton agar; BHI, brain heart infusion broth; NA, nutrient agar medium.

**Table II:** Summary of the inhibitory properties of punicalagin against various viral substances.

Virus	Identifier	IC_50_ (µM)	CC_50_ (µM)	EC_50_	SI	(Refs.)
SARS-CoV-2	293T-ACE2		605.9±61.13	3.70±2.24	163.76	(68)
	NCL-H460		88.43±6.76	4.65±0.47	19.02	(68)
	NSP13 helicase	0.43				(69)
	3CL-protease	6.192^a^				(70)
	3CL^pro^ protease	4.62±0.27				(71)
	Plaque formation		≥100	7.20±1.08		(71)
FHV-1			3.32±0.28			(72)
	RT-associated RNase	0.12±0.00				(73)
	Integrase LEDGF-Dependent	0.065±0.00				(73)
Dengue	NS2B-NS3 protease DENV1	0.91±0.10				(32)
	NS2B-NS3 protease DENV2	0.75±0.05				(32)
	NS2B-NS3 protease DENV3	0.42±0.03				(32)
	NS2B-NS3 protease DENV4	1.80±0.16				(32)
MAYV		29.9±0.9	425±12.8			(74)
IAV	PR8-PB2-Gluc (replication)	1.25±0.06	>>100		>80.0	(75)
	PR8	3.98^a^			6.1	(76)
HCMV	HEL		299.32±9.14	16.76±0.88	17.86	(67)
HCV	Huh-7.5		222.61±3.41	16.72±2.55	13.31	(67)
DENV-2	Vero		151.44±9.31	7.86±0.40	19.27	(67)
MV	CHOSLAM		283.76±11.54	25.49±2.94	11.13	(67)
RSV	HEp-2		264.83±23.71	0.54±0.04	490.43	(67)
VSV	A549		318.84±4.99	36.98±4.59	8.62	(67)
ADV-5	A549		318.84±4.99	196.67±20.05	1.62	(67)
HSV-1	A549		318.84±4.98	10.25±1.13	31.11	(77)
/	MDCK		24.24^a^			(76)

^a^µg/ml. IC_50_, 50% inhibition concentration; CC_50_, 50% cytotoxic concentration; EC_50_, 50% effective concentration; SI, selectivity index; SARS-CoV-2, severe acute respiratory syndrome coronavirus 2; FHV-1, feline herpesvirus-1; HCMV, human cytomegalovirus; HCV, hepatitis C virus; DENV-2, dengue virus 2; MV, measles virus; RSV, respiratory syncytial virus; VSV, vesicular stomatitis virus; ADV-5, adenovirus type-5; HSV-1, herpes simplex virus type-1; MAYV, Mayaro; IAV, influenza A virus.

## Data Availability

Not applicable.
